# Who calls the shots in tobacco control policy? Policy monopolies of pro and anti-tobacco interest groups across six European countries

**DOI:** 10.1186/s12889-019-7158-6

**Published:** 2019-06-21

**Authors:** Thomas G. Kuijpers, Anton E. Kunst, Marc C. Willemsen

**Affiliations:** 1 Department of Health Promotion, CAPHRI Care and Public Health Research Institute, Maastricht University, Peter Debyeplein 1, 6229 HA Maastricht, The Netherlands; 2 Trimbos Institute, Netherlands Institute of Mental Health and Addiction, Utrecht, The Netherlands; 3 Department of Public Health, Academic Medical Center, University of Amsterdam, Amsterdam, The Netherlands

**Keywords:** Policy monopoly, Interest groups, Tobacco control, Group-government relationships, Framing, Institutions, Cross-national

## Abstract

**Background:**

One of the factors influencing variation in tobacco control policies across European countries is the relative policy dominance of pro and anti-tobacco control interest groups. Scholars investigating this power balance have predominantly conducted single country case studies. This study aims to explore and describe the relative dominance of pro and anti-tobacco control interest groups across six European countries by using a tobacco display ban as a case study. We examined whether there are patterns and similarities with regards to two components of policy monopolies: framing of tobacco and institutional arrangements.

**Methods:**

Thirty-two semi-structured interviews with 36 key stakeholders were conducted in Belgium, Finland, Germany, Ireland, Italy, and the Netherlands. These interviews were coded using the Framework Method.

**Results:**

In countries where health Non-Governmental Organizations (NGOs) have a relative policy dominance, tobacco consumption was predominantly framed as a health issue, NGO communities were well developed, the industry was largely absent in terms of production and manufacture, the health ministries played central roles in the policymaking process, and FCTC article 5.3 was strictly interpreted. In countries where the tobacco industry has a relative policy dominance, tobacco was framed as a private problem, NGO communities were absent or weak, the industry was well represented, the health ministries played subordinate roles in the policymaking process, and FCTC article 5.3. was only interpreted in terms of transparency.

**Conclusion:**

The ways in which tobacco consumption is framed in a country and the ways in which institutions are arranged correspond to the policy monopoly in place, with strong similarities across countries with the same policy monopoly.

**Electronic supplementary material:**

The online version of this article (10.1186/s12889-019-7158-6) contains supplementary material, which is available to authorized users.

## Background

Tobacco consumption causes 700,000 deaths per year in the European Union [1]. A recent study in 126 countries investigated the effectiveness of five key tobacco control policy measures and concluded that countries fully implementing more measures experienced greater reductions in smoking prevalence [2]. Tobacco control policy development in European countries is a functioning example of multilevel governance, as policy is developed at various levels [3]. The international level of governance includes efforts by the World Health Organization through the Framework Convention on Tobacco Control (FCTC) and by the European Union through Tobacco Product Directives (TPDs), decisions, regulations, and recommendations. Alongside these international efforts, much of the responsibility for comprehensive tobacco control policy rests with national governments [4].

There are many different ways for national governments to reduce tobacco consumption, including tobacco taxation, smoke free legislation, health warnings, bans on advertising, promotion, and sponsorship and cessation programs [5]. A display ban of tobacco products at points of sale is part of Article 13 of FCTC and is seen as an emerging intervention [6]. European countries demonstrate considerable variation with regard to the implementation of this measure, as some national governments have implemented it more than a decade ago, while others have only recently began to prepare legislation or have not yet begun to discuss it. It is therefore well suited to be a case for a cross-national comparison of tobacco control policymaking.

In explaining variations in tobacco control policy, several theories may be used: 1) policy learning and diffusion theory, 2) theories focusing on the importance of political cultures (e.g. corporatism), 3) theories looking at aspects of institutionalism (e.g. federalism) and 4) theories which focus on the role of interest groups [7]. While all of these theories may offer unique insights into how tobacco control policies are adopted, a growing body of policy research focuses on interest groups and their relative influence on the policy process [8, 9]. Advocacy by interest groups is an important concept in explaining achievements in tobacco control [10, 11]. Stronger regulations are readily attributed to the existence and activities of a relatively strong national network of health NGOs [10, 12, 13] and weaker or averted regulations are attributed to a relatively more dominant tobacco industry and associated businesses [14–16]. It is claimed that without efforts from health NGOs, tobacco control policymaking remains in the hands of policy elites who are susceptible to economic arguments from the tobacco industry [11].

Empirical evidence on the relative power balance of pro and anti-tobacco interest groups is often based on single country case studies [3]. Such studies are able to offer thick and rich descriptions of what is relevant in those countries, providing ‘illuminating accounts of who did what to whom and when’ [4]. However, to better understand differences in tobacco control policy comprehensiveness across political systems, a cross-national approach is preferred [4]. As (political) institutions differ between European countries, a comparison of various political systems can highlight the role of such institutions in the policy process [17]. Single country case studies typically treat such variables as constants [18].

Policy dominance refers to a relative dominance in the process of policymaking of some interest groups rather than others. Although it is acknowledged that interest groups are clashing on an ongoing basis over time to advance their agendas [19], one interest group usually has more power than the other(s) within a given country. This relative dominance can be examined by drawing from the theory of policy monopolies. This interest group theory is well suited for a cross-national comparison, as it allows for the incorporation of framing and (political) institutions, which are both associated with the relative power of pro and anti-tobacco interest groups. A policy monopoly is defined as ‘a monopoly on political understandings concerning the policy of interest and an institutional arrangement that reinforces that understanding’ [20]. A policy monopoly has two main components: 1) the dominant frame of a policy issue and 2) how institutions are arranged to reinforce a certain monopoly [21].

The first component refers to the political understanding of the issue (i.e. the dominant frame). It is argued that only one side of a complex policy issue tends to dominate the public and political discourse at a time, which has an effect on resultant policy outcomes [20]. Often, only a single dimension of a multi-dimensional policy issue gains prominence in the political and public debate [11]. In tobacco control, proponents and opponents of stricter legislation frame the issue of tobacco consumption in different ways, focusing on different dimensions of the policy issue [10]. A relative policy dominance of the tobacco industry and associated businesses may be reflected by liberal-conservative policy frames which highlight positive dimensions of the policy issue, such as the economic benefits of tobacco consumption, employment associated with the tobacco sector, or free individual choice. A relative dominance by health NGOs, on the other hand, may be reflected by policy frames highlighting the negative aspects of the policy issue, focusing predominantly on the detrimental health effects of smoking.

The second component of policy monopolies refers to institutional arrangements, as policy monopolies are hypothesized to be institutionally reinforced [9, 22]. Institutions may be defined as ‘relatively enduring features of political and social life that structure behavior and that cannot be changed easily or instantaneously’ [23]. Institutions are typically created and/or reorganized during short periods of increased attention to a policy issue and remain in place after the attention is directed to other issues, sustaining procedures and biases ‘designed to achieve one set of goals rather than another’ [21]. Examples of institutional arrangements relevant to the power interest groups have in tobacco control are how such groups are organized and a government’s interpretation of FCTC’s Article 5.3 which states that all signed parties ‘act to protect policies from commercial and other vested interests of the tobacco industry in accordance with national law’ [24].

By looking at the case of a tobacco display ban, we will investigate how the countries under study differ with regard to the relative policy dominance of pro and anti-tobacco interest groups. We will focus on the two main components of policy monopolies: the dominant frame of tobacco and institutional arrangements that reinforce a certain monopoly. We will describe and compare policy monopolies of pro or anti-tobacco control interest groups across six European countries.

## Methods

### Project background

This study was part of a larger study conducted in seven EU countries: Belgium, Finland, Germany, Ireland, Italy, the Netherlands, and Portugal. The SILNE-R project aims to assess how smoking prevention strategies are adopted and implemented within seven countries, at national, municipal, and schools levels, and how the process of adoption and implementation varies between countries, cities, and schools.

### Stakeholder selection

National representatives of the SILNE-R project provided a list of key stakeholders relevant to national tobacco control policymaking, in some cases with help of national key informants known to the project. Thirty-four semi-structured interviews with key stakeholders were conducted in English, German, and Dutch. Stakeholders were selected because of their involvement in the tobacco control policymaking process in each country. To get different perspectives on tobacco control policymaking, at least five different types of national level key stakeholders were selected: a civil servant, a member of parliament, an academic expert, an employee of a national cancer fund or other health NGO and, if applicable, an employee of a national tobacco control alliance (see Table 1 for the list with stakeholder professions).

**Table 1:** List of stakeholders per country

Country	Stakeholder function(s)
Belgium	1. Civil servant
	2. Member of parliament (opposition)
	3. Cancer fund employee
	4. Academic expert
	5. Academic expert
	6. Civil society organization employee
Finland	1. Civil servant
	2. Member of parliament (opposition)
	3. Cancer fund employee
	4. Academic expert
	5. Tobacco Control Alliance network employee
	6. Enforcement agency employee
Germany	1. Civil servant
	2. Member of parliament (coalition)
	3. Assistant of member of parliament
	4. Cancer fund employee
	5. Academic expert
	6. Civil society organization employee
	7. Civil society organization employee
Ireland	1. Civil servant
	2. Member of parliament (senate)
	3. Cancer fund employee
	4. Academic expert
	5. Alliance network employee
Italy	1. Civil servant
	2. Civil servant assistant
	3. Cancer fund employee
	4. Academic expert
	5. Academic expert
	6. Civil society organization employee
	7. Civil society organization assistant
The Netherlands	1. Member of parliament (opposition)
	2. Cancer fund employee
	3. Academic expert
	4. Tobacco Control Alliance network employee
	5. Tobacco Control Alliance network employee

A total of 55 stakeholders were contacted for an interview via e-mail, of which 11 did not respond and 10 declined. Non-response was mostly observed from members of parliament. Provided reasons not to participate were either having other obligations or a heavy workload. Thirty-four interviews with 38 stakeholders (i.e. four interviews with two stakeholders per interview) were conducted between January 2017 and August 2017. Twenty-nine interviews were done face-to-face and 5 were done by phone. The interviews lasted 64 min on average and were transcribed verbatim. Each type of stakeholder was successfully interviewed in every country, except for a Dutch civil servant (because of the salience of the policy issue at that time) and an Italian member of parliament (four members of parliament did not respond to the first invitation, nor the reminder).

Portugal was excluded from the study due to continued non-response of stakeholders. We were only able to conduct two phone interviews in Portugal and although these were rich in information, we decided more data was needed to make valid claims about the Portuguese policy process surrounding a tobacco display ban.

### Confidentiality

To ensure confidentiality, we anonymized stakeholder professions as much as possible, on condition that these should not lead to the identification of individuals. Quotes were taken over literally from the transcripts, although we did not select quotes that could lead to identification of specific stakeholders.

### Interview topics

The interviews started with an open question about the current status of a tobacco display ban in the country. Following this question, the first author used a topic list (see the Additional file 1) to bring up various themes: organization of pro and anti-tobacco control interest groups (types of organizations, resources, reasoning, framing, beliefs, priorities, strategies, influence); governmental framing of tobacco consumption; government ideology; country specific themes; access to policymaking (informal rules, FCTC 5.3); administrative capacity; public support; tobacco industry presence; policy learning; and interaction with other policies. The interviewer encouraged spontaneously emerging themes.

### Analysis

The framework method was employed because this methodology allows researchers to analyze the data both by groups of cases (e.g.: individual countries) and by themes [25]. A codebook was developed by coding the Finnish interviews and by subsequently coding the German interviews. The large contrast between these two countries in terms of tobacco control policymaking facilitated the development of a comprehensive codebook. Themes were developed both inductively and deductively, as the main codes (framing and institutions) were theoretically informed and sub-codes were predominantly informed by the interviews.

Informed by the two components of policy monopolies, three main themes were formulated: 1) the dominant frame of tobacco consumption, 2) civil and business institutions (i.e.: businesses such as retailers and the tobacco industry in terms of manufacture and production, since these institutions also affect the ability of the pro-tobacco interest groups to obtain a relative policy dominance), and 3) government institutions. The codebook was further refined and improved by means of multiple discussions with the second and third author. TGK reread the transcripts various times to ensure no themes were missed after modifications to the codebook in later stages. MCW read several transcripts to check for coding rigor, allowing for further refinement of the coding criteria. TGK then systematically coded the complete set of transcripts using MAXQDA version 12 [26]. TGK developed a framework matrix per code, in line with the Framework Method. These matrices contained all coded text segments and were grouped per country. A country summary was made per code. The matrices were checked by MCW as well. The final codebook can be seen in Table 2.

**Table 2:** Overview of codes

Main codes	Code	Sub code
Dominant frame	Public health	- Tobacco as an addictive substance
		- Need to protect children’s health
		- Economic burden to society
	Liberal-conservative	- Smoking as individual choice
		- Tobacco is a legal product
		- Nanny state/patronizing government
	No frames/discussion	
Civil and business institutions	Health advocacy institutions	–
	Retailers	–
	Tobacco industry	- Industry advocacy
		- Industry image
		- Economic presence (Manufacture and production)
Government institutions	Public health policy frameworks	–
	Interpretation FCTC article 5.3	–
	Health ministry centrality	–

## Results

Overall, three clusters of countries emerged from the data: a policy monopoly by health groups in Finland and Ireland; a policy monopoly by the tobacco industry and associated businesses in Germany and Italy; and Belgium and the Netherlands had more complicated policy contexts, as they demonstrated elements both indicative of health and industry monopolies. Table 3 provides a summary of all findings and smoking prevalence per country.

**Table 3:** Overview of findings and smoking prevalence per investigated country

Country	Policy monopoly	Display ban imple-mented	Frame	Health advocacy institutions	Retailers	Tobacco industry economic presence	Public health policy frameworks	Inter-pretation FCTC 5.3	Health ministry role	Smoking pre-valence^a^
Finland	Health	Yes	Health	Developed	Opposition	Largely gone	Endgame strategy	Strict	Central	20%
Ireland	Health	Yes	Health	Developed	Opposition	Largely gone	Endgame strategy	Strict	Central	19%
The Netherlands	Unclear	No	Individual choice/ paternalistic government	Developed	Opposition	Largely gone	No	Strict	–	19%
Belgium	Unclear	No	Individual choice/ paternalistic government	Developed	Opposition	Largely gone	No	Transparency	–	19%
Germany	Tobacco industry and business	No	Private problem/ no discussion	Weak	–	Manufacture and production	No	Transparency	Subordinate	25%
Italy	Tobacco industry and business	No	Private problem/ no discussion	Absent	–	Manufacture and production	No	Transparency	Subordinate	24%

^a^Based on Eurobarometer (2017) item: *‘Do you smoke?’* [1]

### Dominant frame

The dominant government frame refers to how policymakers within individual countries frame the issue of tobacco consumption. Our data suggested that in countries with a health policy dominance (Finland and Ireland), tobacco consumption was predominantly framed as a public health issue, and in countries with a policy dominance by industry and business groups (Germany and Italy), tobacco consumption was mostly framed as a private problem to be dealt with in the private sphere (i.e.: as opposed to a public health problem). Since a policy discussion about tobacco consumption is mostly absent in these two countries (see section below), it can be argued that tobacco consumption is not necessarily considered a policy problem, but rather a private problem for citizens to solve themselves.

In Belgium and the Netherlands, stakeholders indicated that members of the ruling liberal-conservative parties frame tobacco consumption as individual choice and do not want the government to be paternalistic. These frames are similar to the frames used in Germany and Italy, yet in the Netherlands and Belgium, stakeholders explicitly linked these frames to members of the ruling liberal-conservative parties. In Germany and Italy, the reluctance to interfere in ‘private matters’ seemed more wide-spread, crossing both party lines and policy domains.

*‘There was an absolutely unanimous agreement that this is a harmful product. That we are dealing with an industry that has been not just deceitful but has told lies in the past about their knowledge about the damage their product did. And that our government has a duty to protect our children.’*

Ireland, Member of Parliament.

*‘It’s something that is only very reluctantly done in Germany - to have a policy that really influences personal freedom of decision making. So Germany has been very reluctant to do something like that. Not only in health but also in other policies.’*

Germany, civil society advocate.

*‘Germany in particular is very similar to Italy, I think. They are very interested in environmental problems, but the behaviors linked to health are something more personal.’*

Italy, civil society advocate.

*‘The VVD [liberal-conservative ruling party] is an anti-paternalistic party, and tobacco control policies are seen as paternalistic’*

The Netherlands, civil society advocate.

*‘The VLD is liberal-conservative and the mentality there is that everyone has to know for themselves what they do when it comes to protecting their health.’*

Belgium, Member of Parliament

### No frames

An emerging theme from our interviews was that there was no policy debate and therefore, no framing. In Italy and Germany, stakeholders said that nothing other than the strictly necessary debates (e.g. the transposition of the European TPD) were held for the last 10 years. If politicians mention tobacco consumption occasionally, they seem to regard it as a minor problem, or at least a private problem to be dealt with in the private sphere.

*‘Smoking is not very high on the agenda generally - it’s not really perceived as a big problem.’*

Germany, civil society advocate

*‘The parliament addresses tobacco problems only if there is some law in discussion. For example the transposition of the directive, or when the taxes change, or when the smoking ban was proposed, or ten years ago the adoption of the Framework Convention on Tobacco Control. But in other periods they don’t have an interest in tobacco control.’*

Italy, civil servant

### Civil and business institutions

#### Health advocacy institutions

In order to push for tobacco control regulations in general and a tobacco display ban in specific, there needs to be dedicated tobacco control advocates in a given country. How well the NGO community is developed logically affects the ability of tobacco control groups to have and maintain policy dominance and be able to advocate for a tobacco display ban. Our data suggested that such groups were well organized and plentiful in Finland and Ireland, with various degrees of cooperation. In contrast, in Germany, such groups were considered weak and in Italy, such groups did not fully crystallize yet.

*‘In Finland there is a really large NGO community. Huge, powerful NGOs - but you also have to realize that most of the NGOs receive public money.’*

Finland, civil society advocate

*‘We don’t have such a strong NGO structure as in many other countries. It’s mostly health organizations and research institutions that deal with diseases like cancer and others. Therefore they see tobacco as a big problem and engage in tobacco control. There are only very few NGOs, very small … not very powerful... With a few exceptions that only focus on tobacco control.’*

Germany, civil society advocate

*‘There is the need of creating a sort of infrastructure where non-governmental organizations, scientists, cancer patient associations, associations of people with heart attacks, go together in order to push for a new law. This does not exist in Italy at the national level, it is not developed.’*

Italy, civil society advocate

In Belgium and the Netherlands, stakeholders indicated that there is a well-organized NGO community and that there is cooperation between individual NGOs. Belgian stakeholders often contrasted their situation with the Dutch situation and stated that coordination between individual NGOs exists to a lesser degree than in the Netherlands and this was believed to be one of the reasons there has not been much policy development with regard to tobacco control over the last years.

*‘We are heading towards a new modus operandi; we’re starting to delineate that now. But implicitly I’m saying that we are not strong enough right now.’*

Belgium, civil society advocate

*‘The idea behind our alliance is that if you work together, you are much more powerful, and it is better to speak with one voice instead of many different voices who all want something else. Concerning lobby, this works quite well, and if we send a letter, we always do that in name of the three big funds.’*

The Netherlands, civil society advocate

#### Retailers

In addition to the tobacco industry, associated businesses such as retailers are institutions that often oppose stricter future tobacco control legislation, and a tobacco display ban in specific.

In all countries, stakeholders indicated that tobacco retailers expressed themselves against display bans. In Italy and Germany, stakeholders mentioned such opposition less, but as simultaneously observed, a political tobacco control debate in these countries was claimed to be largely absent. In all other countries, retailers have voiced strong opposition towards such a ban. One of the factors brought forward by stakeholders which may explain this opposition is sponsorship contracts with the industry, which were mentioned in interviews from Belgium, Ireland, Italy, and the Netherlands. This is a type of income paid by the tobacco industry to retailers to display tobacco products. Small shopkeepers can be especially dependent on such income, as their total revenue is often lower compared to bigger shops or chains. As an example, in the Netherlands, retailers receive on average 10,250 euros per year to display tobacco packs at points of sale [27].

*‘You will not hear the tobacco industry in the media here in Belgium; it is especially the tradespeople who are very active. And why? Because they receive a lot of money from the tobacco industry because of the sponsorship contracts. He who pays the piper calls the tune.’*

Belgium, civil servant

#### Tobacco industry

Similar to how the organization of health groups affect their ability to have and maintain a health policy dominance in individual countries, the tobacco industry and how well it is represented within a country in terms of production and manufacture logically affects its ability to have and maintain an policy dominance and thus exert influence against the adoption of a tobacco display ban and other regulations.

In countries with a presumed policy monopoly by the tobacco industry and associated business (Germany and Italy), stakeholders indicated that the manufacture and production of tobacco still plays an important role in the domestic economy. Germany is the largest exporter of cigarettes in the EU and second in the world [28]. Italy is the largest tobacco grower of all EU countries, producing 25% of total raw European tobacco crops [29]. Moreover, the economic presence of the tobacco industry in Italy is expanding rather than diminishing, as the new Phillip Morris headquarters for IQOS has opened in 2016 in a village near Bologna, promising up to 600 jobs and investing 500 million euros in the Italian economy [30]. The previous Prime Minister Matteo Renzi and other governmental representatives attended the opening ceremony.

*‘Politicians are very not very keen to face tobacco control. We had Renzi before, a prime minister that was promoting new things with tobacco. Italy is the nation where Philip Morris is testing IQOS. [ …*] *He [Renzi] was really proud of this and the tobacco industry did several investments for plants in several locations near Bologna. There was another 500 million euros promised by 2020 for the purchase of Italian tobacco.’*

Italy, academic expert.

In virtually all German federal states, there are tobacco industry representations in terms of production and manufacture [31]. German stakeholders perceived these local representations to be a deliberate tactic by the tobacco industry, enabling a route of influence from the local constituencies to the federal level, advocating against further tobacco control regulations.

*‘In every state they want to have a little location, not very big, but then they have the right to go to the politicians and say, “You must do something for the jobs. Otherwise we will lose the jobs!”’*

Germany, civil society advocate

*‘There are many actors who can approach individual members of parliament in the constituencies. The influence of the industry via the constituencies and individual members of parliament is stronger than via ministries of the federal government itself.’*

Germany, civil servant

Stakeholders from the other countries (Belgium, Finland, Ireland, and the Netherlands), indicated that the economic presence of the tobacco industry in terms of production and manufacture had diminished over time and is currently small or negligible. In Ireland and Finland, stakeholders stated that the tobacco industry suffers from a bad public image. In both countries, it also seemed part of the NGOs’ strategy to demonize the tobacco industry by labelling them untrustworthy, deceitful, or evil.

*‘Tobacco is not so difficult, because we have already been successful in demonizing -and rightly so - I mean, demonizing the tobacco industry. So that is much more straightforward than alcohol lobbying, which is much more difficult.’*

Finland, civil society advocate

*‘I think the NGOs have generated sufficient levels of distrust among the general public around the tobacco industry. There’s no great love for them, they don’t have presence so they don’t provide loads of jobs and factories that you can identify.’*

Ireland, civil servant

### Government institutions

#### Public health policy frameworks

Public health policy frameworks are governmental commitments to specified public health goals incorporated in national legislation and were only observed in countries where health groups had a clear policy dominance. Such frameworks facilitate the adoption of stricter tobacco control legislation, including display bans, to reach such goals.

Stakeholders in Finland and Ireland described the presence of such national public health policy frameworks. Both of these frameworks concerned endgame strategies with a specified goal of a smoking prevalence of less than 5% in a certain year (2030 in Finland, 2025 in Ireland). Furthermore, stakeholders indicated that both countries have an inter-sectoral approach to policymaking, as exemplified by the ‘Health in all Policies’ initiative in Finland [32], and the ‘Healthy Ireland Framework’ in Ireland [33].

*‘The Healthy Ireland Framework is an initiative that is a cross-sectoral initiative that was launched by the prime minister of the country. It has to do with actions across all these different sectors, but also working with community- and voluntary organizations.’*

Ireland, academic expert

#### Interpretation of FCTC’s article 5.3

An example of a formal institutional arrangement is FCTC Article 5.3, which aims to protect public health policymaking from tobacco industry involvement. All six countries in our study have signed and ratified the FCTC and thus signed to commit themselves to the implementation of article 5.3 as well. However, interpretation of this article varies widely across governments. Our data suggested that countries with a policy dominance by health groups (and the Netherlands) tended to interpret this article more strictly than countries with an industry policy dominance (and Belgium), which seemed to interpret it mostly in terms of transparency. Given the fact that the industry wishes to avoid legislation, including display bans, a weak interpretation of article 5.3 logically results in more influence of the tobacco industry and thus less stringent or no tobacco control legislation as an expected result.

In Finland and Ireland, the industry is invited to public consultations or allowed to send in their submissions on policy proposals, but this is considered part of a standard policy process. Stakeholders stated that the industry could voice their opinions, but that there are no further negotiations. A few stakeholders however also stated, that the industry can sometimes come up with useful additions to policy proposals, for example in relation to certain implementation issues. In Ireland, stakeholders indicated that they were met in relation to specific issues, such as commerce and smuggling.

*‘I think in principle if members of the [health] committee say: “I want to listen to the representative of Philip Morris”, then that person will be invited. In the FCTC, there is this famous article 5.3 which says that tobacco companies and tobacco industry must not be involved in tobacco policymaking, and that is very well followed in most of the Western countries, like in Finland. So when the ministry and the government propose legislation, they don’t negotiate with the tobacco industry anymore. The tobacco industry can send a letter to them if they want, but there’s no negotiating anymore.’*

Finland, Member of Parliament

*‘Not that they won’t listen. They listen, assess, and make a decision, in fairness. The WHO though, made it very clear that we shouldn’t be meeting with tobacco companies when we are talking about tobacco policy. It is alright to meet in regard to other matters in relation to commerce and smuggling and all that stuff. That’s fine.’*

Ireland, Member of Parliament

In the Netherlands, although it is not clear whether health groups have a policy monopoly, article 5.3 is strictly interpreted. A stakeholder stated that the ministries of health and finance developed an internal document describing rules of conduct to deal with advocates from the tobacco industry, which was perceived to be the result of a court case from a NGO against the Dutch state. According to the stakeholders, this resulted in an interpretation which includes the industry only when it comes to technical implementation issues and that these contacts need to be transparent. This interpretation is quite similar to the interpretation in Finland and Ireland.

*‘At this moment, the guideline for civil servants is that one should limit oneself to technical implementation issues.’*

Netherlands, academic expert

In the other countries (Belgium, Italy and Germany), stakeholders stated that FCTC’s article 5.3 is predominantly interpreted in terms of transparency, and it was noted that there are no formal rules of conduct for civil servants. In Italy, a stakeholder indicated that ministries other than health seem to take many liberties with regard to their contacts with the tobacco industry, as long as they report all interactions afterwards. In Germany, a stakeholder stated that the ministry of food and agriculture - responsible for tobacco product regulation - reports meetings with the industry on their website, with the subject of the meeting and with whom the meeting was held, but no further information is provided. When these documents are requested by means of a freedom of information act request, they are received with large parts blacked out.

*‘There is a sort of light interpretation because they intend article 5.3 only on the side of transparency. If the relations are transparent, you can do everything.’*

Italy, civil servant

*‘The ministry of food and agriculture says: “We show what meetings we have on our internet site”. But all you see is for example the date and it says the ministry and there were for example [representatives from the] “Deutschen Zigarettenverband” [an organization representing five tobacco manufacturers] and they talked about taxes or something like that, and you don’t get any more information. They say this fulfils 5.3. This is transparent. “Look here: we have showed that we have met with them”. And the names of the people of the government are blacked out. If we do get information, then many things are blacked out.’*

Germany, civil society advocate

#### Health ministry centrality

When the health ministry plays a central role in policymaking, resultant policy is likely stricter and more health oriented than when other ministries such as trade and finance take the policy lead. Our data suggested that countries where there is a policy dominance by health groups (Finland and Ireland), the health ministry played central roles in the policy process, and in countries where the industry has more influence, the health ministry plays a more subordinate role in the process of policymaking (Germany and Italy).

In Finland and Ireland, the ministries of health took the policy lead and introduced new tobacco control initiatives, even in the absence of active advocacy from the health NGOs. This was said to be the case with the development of the previous tobacco acts in both countries, in which tobacco display bans were included as relatively minor issues in a large comprehensive packages of policy measures.

*‘He [health ministry civil servant] often was looking for the NGOs support for what he was doing, than the other way around. I think on many of the issues around some of these things he was very far-reaching and looking hard. So the NGOs were behind him, supportive … He was the author of a lot of the legislation at the time.’*

Ireland, civil servant

*‘Well in Finland we had the working group for what should be done for tobacco policy. It was quite a large-scale working group, led by the ministry of health. [ …*] *They published their report in 2009 and there were many suggestions to improve the Tobacco Act [ …*]. *This [a display ban] was one of those suggestions which was ultimately implemented.’*

Finland, civil servant

In the Netherlands and Belgium, stakeholders said that technically the health ministry has responsibility for tobacco control policy, yet it was further remarked that there was an unwillingness of liberal-conservative ruling parties to regulate any health behaviors. In Belgium, stakeholders remarked that the liberal-conservative Minister of Health seems to explicitly exclude the ministry of health from the policy process, as she predominantly consults a small set of personal staff members and party-loyal political advisors.

*‘This minister relies very heavily on her small entourage and involves the ministry only little. She sometimes even makes decisions without the ministry knowing.’*

Belgium, civil society advocate

In counties with an industry policy dominance (Italy and Germany), stakeholders stated that the health ministry plays a less central role in tobacco policymaking. In Germany, this is very apparent, because the legislative jurisdiction with regard to tobacco policy when it comes to product regulation was said to reside in the federal ministry of food and agriculture. When it comes to prevention issues, the federal ministry of health was said to have jurisdiction. However, when the ministry of health wants to make tobacco control policy, one stakeholder noted that they have to prompt other ministries to prepare it. In Italy, it seems that although officially the health ministry has formal jurisdiction with regard to tobacco control policy, in practice they are perceived to fulfil an underdog position. Other ministries, such as the ministry of agricultural, food and forestry policies, economic development, economy and finance all were, as an illustration, primarily involved with the transposition of the European TPD. The ministry of health was consulted last.

*‘For tobacco and alcohol policy, responsibility in terms of product regulation mainly resides in the food and agriculture ministry. Responsibility for prevention resides in the ministry of health. The health ministry cannot simply say, “We will propose a bill and let’s get it done”. It would be nice, but unfortunately this is not the case.’*

Germany, civil servant

## Discussion

In countries with a similar policy dominance (i.e. more relative influence from either pro or anti-tobacco interest groups), the same dominant frames were adopted, and civil and governmental institutions were arranged in comparable ways. In countries where there was a health policy monopoly, stakeholders indicated that tobacco consumption was framed as an incontestable public health problem, there were many well-developed health NGOs, the tobacco industry was largely gone and publicly discredited, the health ministry played a central role in tobacco control policy development, and FCTC’s Article 5.3 was more strictly interpreted. In these countries, tobacco display bans were adopted more than a decade ago, as parts of a larger comprehensive policy packages. A largely reversed image was observed in countries where there was a tobacco industry policy monopoly. In these countries, stakeholders indicated that tobacco consumption was generally framed as a private problem of citizens, the health NGO communities were weak or absent in the tobacco control area, the tobacco industry still played a role in the domestic economy, while health ministries played subordinate roles in the formation of tobacco control policies, and FCTC’s article 5.3 was primarily interpreted in terms of transparency. In these countries, tobacco control issues, including a display ban, were not discussed in parliament for the last decade, apart from the necessary debates on transposition of European Tobacco Product Directives.

Our findings seem to illustrate an antagonism between pro and anti- tobacco control interest groups, where a relative policy dominance only seems to be maintained due to a lack of interference by opposing interest groups [19]. This was the case in Ireland and Finland, where stakeholders stated there is a well-developed health NGO community and a largely absent (in terms of production and manufacture), and publicly discredited tobacco industry. In these two countries, the health NGOs were perceived to have a prominent role in shaping tobacco control policy.

In strong contrast to Finland and Ireland, stakeholders in Italy and Germany reported a considerable tobacco industry presence and a relatively weak or absent NGO community. This may leave the tobacco control policymaking process more susceptible to the tobacco industry, which may exert their influence through other more powerful ministries, particularly the ministries of trade, finance, and agriculture.

Belgium and the Netherlands may be positioned in between these extremes, having mixed profiles containing elements both indicative of health and industry monopolies. Stakeholders from these countries stated that there is a NGO community in which independent NGOs join forces in advocating for tobacco control policy, but that members of the ruling liberal-conservative political parties are reluctant to impose regulations in the health domain because they are perceived to be paternalistic. This was especially noticeable in Belgium, where the Minister of Health is from a liberal-conservative party and is unwilling to include the ministry of health into the drafting of a new tobacco plan.

When considering these three types of countries, it is illustrative to refer to Young (2006) who makes a distinction between three types of government - non-profits relationships. These relationships can be either complementary (in which the non-profits and government work together in partnership), supplementary (in which goods or services are provided in addition to those provided by the government), or adversarial (in which non-profits urge the government to make changes in public policy) [34]. The complementary type of relationship is most applicable to Finland and Ireland, as there is close cooperation and partnership between NGOs and the government. The supplementary type is more applicable to Belgium and the Netherlands, where the NGOs may or may not be consulted, depending on the current ideology of the ruling parties. The situation in Italy and Germany seems most compatible with the last category, in which demands for change are voiced but do not seem to find much resonance within the government.

Some findings of this study closely resemble factors identified by Cairney & Mamudu (2014) on basis of interviews with more than 300 policy participants across 39 countries. These authors describe ‘ideal type’ policy environments for tobacco control, where the department of health must take the policy lead; tobacco is framed as a public health problem; and the tobacco companies are excluded from the policy process, while consulting public health groups [35]. They also describe the interrelatedness of some of these factors: having a health ministry that plays a central role in the process of policymaking automatically fosters the inclusion of public health groups and the exclusion of tobacco companies. Furthermore, having a central health ministry will likely keep the focus on health aspects of smoking (in contrast to other ministries such as trade and finance). Our findings confirm these factors, and our most progressive countries (i.e. Finland and Ireland) closely resemble their description of ‘ideal type’ policy environments.

This study is consistent with the assumption that national level tobacco control comprehensiveness is related to the relative power balance of national pro and anti-tobacco interest groups, as illustrated by the case of a tobacco display ban. The two countries that had a policy dominance by health groups, Finland and Ireland, were the only two countries in this study to adopt and implement a tobacco display ban in 2010 and 2002 respectively [36, 37]. These bans were considered relatively minor issues in a larger comprehensive package of policy measures. In countries in which the tobacco industry was suggested to have more relative policy dominance (Germany and Italy), there had been no tobacco control debate for the last decade or so, apart from the necessary debates on transposition of the TPD, suggesting policy inertia. In Belgium, a tobacco display ban was proposed within a larger policy package by two members of one of four ruling parties in Belgium in 2016 [38], but did not get a majority of votes in the House of Representatives, which is commonly observed in Belgium for proposals that seek alternative majorities (Keppens & Van Waeyenberg: Wisselmeerderheden in België doorgelicht, unpublished). In the Netherlands, the House of Representatives adopted a motion in 2015, calling on the government to reach a voluntary agreement with supermarkets to implement a display ban [39]. After several attempts, the State Secretary for Health concluded that such a voluntary agreement did not seem possible. In 2017, the parliament voted in favor of a legislative amendment to introduce a display ban [40].

A possible limitation of this study was that all findings rely on the perceptions of a limited number of key stakeholders per country. Although the stakeholders were carefully selected because of their central roles in the tobacco control policy process, their views may not be completely representative of tobacco control policymaking processes in their countries. However, the accounts from different stakeholders within a country demonstrated considerable similarities and compatibility, suggesting that they are indeed representative of the ‘actual’ policymaking processes in these countries.

Furthermore, we acknowledge that the policy processes underlying the variance in tobacco control policy comprehensiveness across different European countries are highly idiosyncratic and subject to numerous influences (e.g. historical, cultural) [4]. However, despite these differences, we would like to emphasize that these countries also demonstrate considerable similarities with regard to framing and institutional arrangements, dependent on the policy monopoly in place.

Finally, the proposition that one of the two interest groups has a relative policy dominance may sound simplistic or even deterministic. Their relative power may better be conceived of as a continuum rather than in a strictly binary sense. The observation that one of the two interest groups has more power than the other within a country at a single point in time, does not automatically suggest that the other group is powerless. Pro- and anti-tobacco interest groups are known to clash on an ongoing basis over time to advance their respective agendas [19].

## Conclusion

This study was the first empirical assessment of the power balance between pro and anti-tobacco control interest groups across six European countries. Findings indicate that both framing and institutional arrangements coincide with the policy monopoly in place and that there are remarkable similarities across countries with the same suggested monopoly. If health advocates want to challenge an industry monopoly to push for more stringent legislation, including tobacco display bans, they may elect to adopt an approach that not only focuses on framing, but also targets the institutional arrangements which reinforce a policy monopoly by the tobacco industry.

## Additional file

Additional file 1: Topic List. (DOCX 25 kb)

## Data Availability

The transcripts and datasets of this study are not publicly available as access would directly contravene obligations to interviewees made as part of the consent process.

## Abbreviations

EU: European Union
FCTC: Framework Convention on Tobacco Control
NGO: Non-Governmental Organization
TPD: Tobacco Product Directive
WHO: World Health Organization
