# Supplementary material for: Who calls the shots in tobacco control policy? Policy monopolies of pro and anti-tobacco interest groups across six European countries
Source: BMC Public Health. 2019 Jun 21;19:800. doi: 10.1186/s12889-019-7158-6 (PMC6589028; doi:10.1186/s12889-019-7158-6)
Supplement: Supplementary file 1 — Topic List. (DOCX 25 kb) [file 12889_2019_7158_MOESM1_ESM.docx]

**Topic List**

| **Main Questions** | **Possible additional questions** | **Clarifying questions** |
| --- | --- | --- |
| 0. Can you tell me something about how the current status of the POS display ban in [country]?  01. Is it discussed in parliament? Why (not?) | 1. How? Why? | Can you tell me a bit more about that?  Can you give an example?  How?  Why?  Can you tell me a bit more about that?  Can you give an example?  How?  Why?  Can you tell me a bit more about that?  Can you give an example?  How?  Why? |
| *Per country there are usually two sides when it comes to tobacco control measures: a side that tries to promote more stringent tobacco control measures (the health-side), and a side that tries to prevent or delay more stringent tobacco control measures (the tobacco side).*  1. What can you tell me about the health side in [country]?  2. What can you tell me about the pro-tobacco side in [country]? | 1. Are they organized? How? 2. Can you tell me something about their resources? E.g. money, size, expertise? 3. What kind of organizations are part of this side? 4. Does the health-side collect data about smoking prevalence and public support? Why? 5. Is there a reasoning behind a POS display ban (both sides?) Arguments? 6. Do all parties at the health-side have the same beliefs about a POS display ban? As a policy solution? 7. Is the POS display ban a priority of the health-side? Why (not)?   7B. What can you tell me about the strategy of the health-side to realize a POS display ban?   1. What can you tell me about the strategy of the tobacco-side to block a POS display ban? |  |
| 2. What can you tell me about the influence of both sides on the policy process surrounding the POS display ban? | 1. Do you think one of the sides exerts more influence on the policy process than the other? How? Why? |  |
| 3. How do NGO’s talk about (frame) a POS display ban? | 1. Does the government adopt one of these frames? Can you give an example? |  |
| 4. How does the tobacco industry talk about (frame) a POS display ban? |  |  |
| 5. What can you tell me about the general ideological outlook of the government when it comes to smoking? | 1. To what extent do you think is related to the influence both sides have on the policy process of the POS display ban? 2. Do you think that ideology plays a role in the policy process? How? |  |
| 6. Do you think there are country-specific characteristics that are of influence on tobacco control in [country]? |  |  |
| 7. To what extent are public parties incorporated in the policy process (of a POS display ban)? | 1. Who has access to the policy process? 2. Are there rules (explicit or implicit) for who can or cannot access the policy process? (FCTC 5.3)      1. Can everybody get access to the policy process? 2. Do you think that one of the two sides has more access to the policy process than the other? |  |
| 8. Can you tell me something about the administrative capacity of the civil servants that work on tobacco? The ministry? | 1. Is there a separate unit that works on tobacco? 2. How many people work on the topic? |  |
| 9. What role does public support play in relation to a POS display ban? |  |  |
| 10. Can you tell me how important the tobacco sector is for the national economy? | 1. Do you think this is related to the influence the tobacco-side has on politics? How? 2. Do you think this affects the progression of a POS display ban? |  |
| 9. Did the government look abroad to other country experiences with a POS display ban? | 1. What countries? Why these countries? |  |
| 10. To what extent do you think other tobacco control policies has had an influence on the adoption of the POS display ban? | 1. What policies? 2. Why these policies? 3. How? |  |
